# Neuroprotective body hypothermia among newborns with hypoxic ischemic encephalopathy: three-year experience in a tertiary university hospital. A retrospective observational study: Hipotermia corpórea neuroprotetora em recém-nascidos com encefalopatia hipóxico-isquêmica: três anos de experiência em hospital universitário terciário. Um estudo observacional retrospectivo

**DOI:** 10.1590/1516-3180.2013.7740026

**Published:** 2014-10-28

**Authors:** Mauricio Magalhães, Francisco Paulo Martins Rodrigues, Maria Renata Tollio Chopard, Victoria Catarina de Albuquerque Melo, Amanda Melhado, Inez Oliveira, Clery Bernardi Gallacci, Paulo Roberto Pachi, Tabajara Barbosa Lima

**Affiliations:** I MD, MSc. Head, Division of Neonatology, Department of Pediatrics, Santa Casa de São Paulo, São Paulo, Brazil.; II MD, PhD. Assistant Professor, Division of Neonatology, Department of Pediatrics, Santa Casa de São Paulo, São Paulo, Brazil.; III MD, MSc. Instructor Professor, Division of Neonatology, Department of Pediatrics, Santa Casa de São Paulo, São Paulo, Brazil.; IV MD. Instructor Professor, Division of Neonatology, Department of Pediatrics, Santa Casa de São Paulo, São Paulo, Brazil.; V MD. Resident, Division of Neonatology, Department of Pediatrics, Santa Casa de São Paulo, São Paulo, Brazil.; VI MD, PhD. Assistant Professor, Division of Neonatology, Department of Pediatrics, Santa Casa de São Paulo, São Paulo, Brazil.

**Keywords:** Infant, newborn, Hypoxia, brain, Hypothermia, Asphyxia neonatorum, Magnetic resonance imaging, Recém-nascido, Hipóxia encefálica, Hipotermia, Asfixia neonatal, Imagem por ressonância magnética

## Abstract

**CONTEXT AND OBJECTIVE:**

Neonatal hypoxic-ischemic encephalopathy is associated with high morbidity and mortality. Studies have shown that therapeutic hypothermia decreases neurological sequelae and death. Our aim was therefore to report on a three-year experience of therapeutic hypothermia among asphyxiated newborns.

**DESIGN AND SETTING:**

Retrospective study, conducted in a university hospital.

**METHODS:**

Thirty-five patients with perinatal asphyxia undergoing body cooling between May 2009 and November 2012 were evaluated.

**RESULTS:**

Thirty-nine infants fulfilled the hypothermia protocol criteria. Four newborns were removed from study due to refractory septic shock, non-maintenance of temperature and severe coagulopathy. The median Apgar scores at 1 and 5 minutes were 2 and 5. The main complication was infection, diagnosed in seven mothers (20%) and 14 newborns (40%). Convulsions occurred in 15 infants (43%). Thirty-one patients (88.6%) required mechanical ventilation and 14 of them (45%) were extubated within 24 hours. The duration of mechanical ventilation among the others was 7.7 days. The cooling protocol was started 1.8 hours after birth. All patients showed elevated levels of creatine phosphokinase, creatine phosphokinase-MB and lactate dehydrogenase. There was no severe arrhythmia; one newborn (2.9%) presented controlled coagulopathy. Four patients (11.4%) presented controlled hypotension. Twenty-nine patients (82.9%) underwent cerebral ultrasonography and 10 of them (34.5%) presented white matter hyper-echogenicity. Brain magnetic resonance imaging was performed on 33 infants (94.3%) and 11 of them (33.3%) presented hypoxic-ischemic changes. The hospital stay was 23 days. All newborns were discharged. Two patients (5.8%) needed gastrostomy.

**CONCLUSION:**

Hypothermia as therapy for asphyxiated newborns was shown to be safe.

## RESUMO

**CONTEXTO E OBJETIVO:**

A encefalopatia hipóxico-isquêmica neonatal apresenta alta morbi-mortalidade. Estudos com hipotermia comprovam diminuição de sequelas neurológicas e morte. Nosso objetivo foi então relatar experiência de três anos da hipotermia terapêutica em recém-nascidos (RN) asfixiados.

**TIPO DE ESTUDO E LOCAL:**

Estudo restrospectivo, conduzido em hospital universitário.

**MÉTODOS:**

Trinta e cinco pacientes com asfixia perinatal submetidos a resfriamento corporal entre maio de 2009 e novembro de 2012 foram avaliados.

**RESULTADOS:**

Trinta e nove RN preencheram os critérios do protocolo de hipotermia. Quatro RN foram excluídos devido a choque séptico refratário, não manutenção da temperatura e coagulopatia grave. A mediana do Apgar de 1^o^ e 5^o^ minutos foi de 2 e 5. A maior complicação foi infecção, diagnosticada em sete mães (20%) e 14 RN (40%). Convulsão ocorreu em 15 RN (43%). 31 pacientes (88,6%) necessitaram da ventilação mecânica e 14 (45%) foram extubados em 24 horas. O tempo de ventilação mecânica dos demais foi de 7,7 dias. O início do resfriamento ocorreu com 1,8 horas de vida. Todos os pacientes apresentaram níveis elevados de creatinofosfoquinase, creatinofosfoquinase-MB e desidrogenase lática. Não se observou arritmia grave; um RN (2,9%) apresentou coagulopatia controlada. Quatro pacientes (11,4%) tiveram hipotensão controlada. Realizou-se ultrassonografia cerebral em 29 pacientes (82,9%), 10 (34,5%) com hiperecogenicidade da substância branca. 33 RN (94,3%) fizeram ressonância magnética cerebral, 11 (33,3%) com alterações hipóxico-isquêmicas. O tempo de internação foi de 23 dias e todos receberam alta. Dois pacientes (5,8%) necessitaram de gastrostomia.

**CONCLUSÃO:**

A hipotermia como terapêutica para RN asfixiados demonstrou ser segura.

## INTRODUCTION

Perinatal asphyxia consists of decreased metabolic and nutritional intake from mother to fetus, thereby causing low fetal tissue perfusion, hypoxia, hypercapnia and acidosis. One of its main consequences is hypoxic-ischemic encephalopathy (HIE), which occurs in one to three cases per 1000 full-term newborns.^1^^-^^2^ The outcomes from HIE range from intact survival to death. The spectrum of long-term morbidity among survivors ranges from mild motor and cognitive deficits to cerebral palsy and severe cognitive deficits.^3^ It used to be believed that HIE was caused by intrapartum events and umbilical cord prolapse, breech presentation, forceps delivery and maternal fever.^4^ However, intrapartum factors have been found to be the cause of HIE in only 4% of affected newborns, while 69% of such cases have shown evidence of antenatal risk factors such as severe preeclampsia, maternal thyroid disease, viral infection, moderate to severe vaginal bleeding during pregnancy and maternal hypertension.^5^^,^^6^ Restriction of primary and secondary intrauterine growth has also presented a strong association with HIE.^4^ However, little evidence of antenatal damage has been observed on brain magnetic resonance imaging (MRI) performed at an early stage on neonates suffering from HIE.^7^

Until recently, clinical HIE treatment consisted basically of neonatal intensive care support, correction of metabolic respiratory and hemodynamic disorders and use of anticonvulsants. However, studies published over the last eight years have individually or collectively shown the effectiveness of using body hypothermia for HIE treatment, thereby promoting increased survival without neurological sequelae, with lower morbidity and mortality.^8^^,^^9^

Since the time of Hippocrates, therapeutic hypothermia has been applied to various clinical conditions. In a book written by Sir John Floyer, a physician and writer in the 17^th^ century, a procedure in which a stillborn infant was immersed in cold water to induce spontaneous breathing is described.^10^ The history of modern science has included periodic attempts to standardize the use of therapeutic hypothermia for a range of cerebral injury; this movement has been accelerated by advances in cardiopulmonary resuscitation.^10^^,^^11^ Randomized controlled trials on the effectiveness of therapeutic hypothermia in adult patients after cardiac arrest have shown improvements in survival and neurological outcomes.^12^

Hypothermia reduces brain injury through its impact on several biological processes. It reduces vasogenic edema, hemorrhage and neutrophil infiltration. It limits the release of excitatory neurotransmitters and the accumulation of intracellular calcium. The production of free radicals is restricted by hypothermia, and thus cells and organelles are protected from oxidative damage during reperfusion. Also, it reduces the activation of cytokine and coagulation cascades by increasing the concentration of interleukin-10, an anti-inflammatory cytokine, and reducing tumor necrosis factor-alpha. Furthermore, hypothermia helps to maintain cerebral metabolism during and after cerebral attacks by decreasing the metabolic rate of glucose and oxygen. Through reducing caspase-3 activity and increasing the expression of the anti-apoptotic protein BCL-2, hypothermia limits neuronal apoptotic death.^13^

Among newborns, this therapy consists of reducing body temperature by three to four degrees Celsius (moderate hypothermia), starting within six hours of birth and continuing for 72 hours. The efficacy and safety of this treatment have been confirmed in other studies and meta-analyses, and this has led to the introduction of hypothermia therapy protocols in daily clinical practice in many neonatal units worldwide.^14^^,^^15^^,^^16^ Our neonatology service has been using hypothermia as a routine clinical therapeutic practice since 2009 and was the pioneer in introducing a hypothermia protocol to Brazilian neonatal units, with effective participation by all the professionals involved in high-risk newborn care, from birth in the obstetric delivery room to procedures in the neonatal intensive care unit.

## OBJECTIVE

Our aim in this study was therefore to report on our experience of three years of hypothermia therapy on asphyxiated newborns in a tertiary university hospital, demonstrating the characteristics of the newborn population undergoing this therapy, birth conditions, clinical complications, adverse effects, features of body temperature control and follow-up during the stay in the neonatal unit.

## METHODS

**Chart 1.**
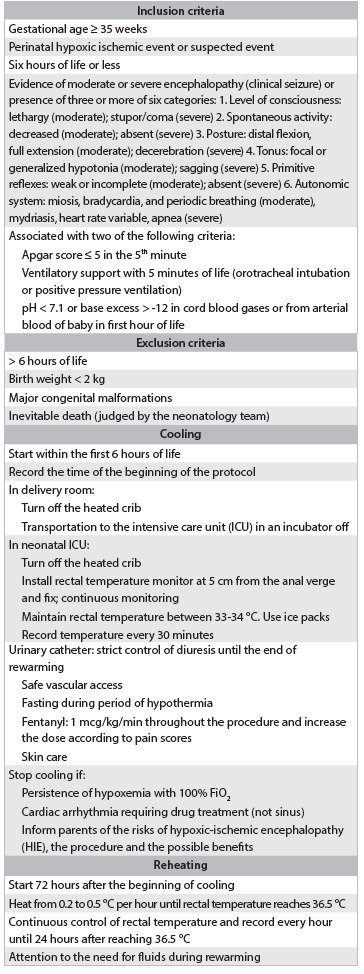
Total body cooling protocol for infants with hypoxic-ischemic encephalopathy

This was a retrospective observational study on a cohort of newborns with HIE who fulfilled the criteria for inclusion in a total body cooling protocol at a neonatology service in a tertiary-level university hospital (Chart 1) and were treated between May 2009 and November 2012. The laboratory tests indicative of perinatal asphyxia comprised gas analysis (pH and base excess, BE) on samples collected from cord blood or from the newborn within the first hour of life and assays on the enzymes creatine phosphokinase (CPK), creatine phosphokinase-MB (CK-MB) and lactate dehydrogenase (LDH) collected from the infant before the sixth hour of life. Furthermore, we analyzed the time required to reach the target temperature of 34 °C. The possible adverse effects evaluated were cardiac arrhythmias, hypotension, coagulopathy, infection and death.

We performed the following imaging studies: cerebral ultrasonography (US) on first day of life and brain computed tomography (CT) and/or magnetic resonance imaging (MRI) between the 5^th^ and 21^st^ days. We took into consideration changes in the analyses that were consistent with hypoxic-ischemic injury.

We used the following assessments to determine the final outcome during the hospitalization period: length of hospital stay, mortality and need for gastrostomy.

For the statistical analysis, we firstly performed descriptive analysis on the data. Qualitative variables were represented as absolute and relative frequencies. Numerical variables were expressed as mean, median, standard deviation (SD) and minimum and maximum values. In cases in which we found great variability in the samples, we chose to use the median. We used the chi-square test to compare qualitative variables and the nonparametric Kruskal-Wallis test to compare the Apgar variable. P-values (P) ≤ 0.05 were considered statistically significant. We used the Minitab-15 statistical software.

The study was approved by our institution’s Ethics Committee for Human Research.

## RESULTS

Thirty-nine infants met the criteria for inclusion in the hypothermia protocol during the study period. Four of them had to be withdrawn from the study: two developed refractory septic shock, one failed to maintain a temperature below 34 °C and one evolved with severe coagulopathy. The perinatal characteristics of the 35 patients studied are shown in Table 1.

**Table 1.**
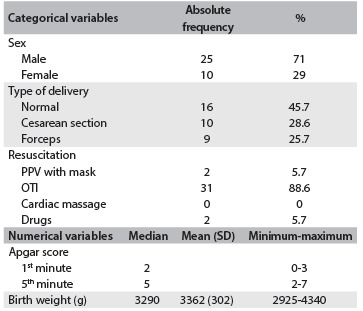
Perinatal characteristics of patients

PPV = pressure ventilation; OTI = orotracheal intubation; SD = standard deviation.

With regard to the type of delivery (vaginal or cesarean), we did not find any statistically significant difference between the sexes (P = 0.42). Comparing the type of delivery with the Apgar scores at 1 and 5 minutes, there were no statistical differences (P = 0.096 and 0.287). There were no statistical differences in the Apgar scores at 1 and 5 minutes in relation to sex (P = 0.847 and 0.296).

Nine pregnant women (25.7%) had no complications during pregnancy. The main maternal complication was infection, diagnosed in seven patients (20%). Infection was also the most commonly diagnosed complication in the newborns, found in 14 of them (40%). Five of these (35.7%) had positive blood cultures. Seizures were diagnosed in 15 newborns (43%): 11 of them (73.3%) received monotherapy treatment (phenobarbital) and four (27%) received diphenylhydantoin in association with phenobarbital.

Thirty-one patients (88.6%) required mechanical ventilation and 14 (45%) of these patients were extubated within 24 hours of life. Among the other 17 infants, the duration of mechanical ventilation was 7.7 days (SD = 5.6), with a median of six days.

Body cooling began on average 1.8 hours (SD = 1.8) after birth (median of 1 hour). The target temperature was attained, on average, 1.2 hours after the beginning of the protocol, and 21 infants (60%) achieved this temperature within 1 hour.

All patients from whom CPK, CK-MB and LDH enzyme samples were obtained showed elevated levels. There was only one patient without enzyme samples. The initial laboratory abnormalities are shown in Table 2.

**Table 2.**
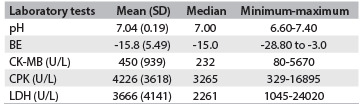
Laboratory abnormalities

BE = base excess; CPK = creatine phosphokinase; CK-MB = creatine phosphokinase-MB; LDH = lactate dehydrogenase.

Considering the possible adverse effects resulting from hypothermia, no patient had any arrhythmia other than sinus bradycardia and only one patient (2.9%) had coagulopathy requiring intervention (Table 3). Four (11.4%) of the initial 35 newborns presented hypotension requiring vasoactive drugs.

**Table 3.**
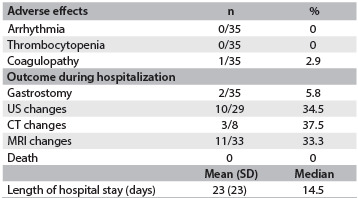
Adverse effects and outcome during hospitalization

US = Ultrasonography; CT = brain computed tomography; MRI = magnetic resonance imaging.

The cases prior to March 2011 (16 children) underwent ultrasonography and/or computed tomography (CT) and two cases did not undergo magnetic resonance imaging (MRI) before discharge. From March 2011 onwards, all the patients underwent MRI. The data relating to the hospital outcomes are reported in Table 3. Cerebral ultrasonography was performed on 29 patients (82.9%): 19 (65.5%) were normal and 10 (34.5%) had hyperechogenicity of white matter. Brain MRI was performed on 33 infants, and 11 of them (33.3%) had signs suggestive of hypoxic-ischemic encephalopathy.

All the infants were discharged from the neonatal unit. Only two patients (5.8%) were discharged with gastrostomy.

## DISCUSSION

This study reports on a three-year experience of administering hypothermia therapy to asphyxiated newborns in a tertiary-level university hospital. The criteria for infant inclusion and exclusion were based on previous studies on safety. The gestational age for inclusion in the study (more than 35 weeks) made it possible to differentiate encephalopathy attributed to perinatal hypoxia from other problems relating to prematurity.^17^ Hypothermia is applied within the first six hours of life because this is the therapeutic window within which the neuroprotective effect relating to reduction of cerebral metabolism, reduction of excitatory neurotransmitter activity, suppression of free radical release, inhibition of the apoptotic process and reduction of the release of inflammatory mediators is most effective. In general, the efficacy of the neuroprotective effect diminishes if the cooling period starts after the therapeutic window, but evidence suggests that the neurological injury in HIE cases continues beyond this period.^18^

The fact that no significant complications attributed to therapeutic hypothermia were observed, with successful maintenance of body temperature within 72 hours, shows that this therapy is safe and easy to control and maintain when a multidisciplinary team is involved with the main aim of reducing neurological sequelae among infants with HIE. Regarding sedation, given that it is difficult to determine the degree of pain or discomfort in newborns with HIE who undergo hypothermia, and that opioids appear to boost the neuroprotective effect of this therapy, we considered that it would be beneficial to use systematic sedation with low doses of opioids in this group of infants.^9^

All the patients with HIE who underwent hypothermia were kept fasting for 72 hours, in accordance with the protocols of previously published clinical trials.^8^^,^^9^^,^^10^^,^^11^^,^^12^^,^^13^^,^^14^ Hypothermia could increase the already-present risk of occurrences of necrotizing enterocolitis secondary to intestinal ischemia and hypoxia. This approach contributed towards the absence of gastrointestinal complications in our patients, as also seen in the results from the abovementioned studies.^8^^,^^9^

Although we used cold packs rather than thermal mattresses, we did not record any difficulties in maintaining the temperature within the established goals. Use of gel packs has been reported to reduce death or developmental delays at six months of age among infants with HIE, without increasing the adverse events,^19^ although use of servo control systems can avoid temperature fluctuations.^20^ Moreover, we had no difficulties during reheating.

We did not find any serious side effects or complications that were directly related to hypothermia. As in other studies,^9^^,^^10^^,^^11^^,^^12^^,^^13^^,^^14^ we had cases of sinus bradycardia without hemodynamic effects. Cooling induces QTc prolongations in hypoxic full-term newborns that become normal with rewarming.^21^ In our study, no patient had severe arrhythmia requiring antiarrhythmic intervention.

We had clinical diagnoses of seizures in 15 infants, representing 43% of the study group. Electrographic seizures are common and often nonconvulsive, and their onset occurs over a broad range of times within the first days of life.^22^ Unfortunately, amplitude-integrated electroencephalograms (aEEGs) available for continuous electroencephalographic monitoring were not available to us. aEEGs might have provided greater accuracy in diagnosing seizures, since it is known that asphyxiated newborns can present neuronal hyperexcitability without evidence on clinical examination. The changes found on brain US are not specific to HIE, but changes seen on MRI are more reliable, especially for demonstrating changes to the basal ganglia and thalamus. The development and severity of motor deficits due to perinatal asphyxia correlate with the pattern of brain injury seen on MRI. MRI provides valuable prognostic information on hypothermia-treated infants.^23^ There may be a selective neuroprotective effect on the cerebral cortex from hypothermia, with improved neurocognitive functioning.^24^ The ideal time to perform MRI is between the fifth and eleventh days of life, because the imaging findings have a better relationship to the neurological prognosis.^25^ We observed seizures in only 43% of the newborns, and there were no deaths, which may have demonstrated that the profile of the group studied was more similar to patients with moderate HIE. However, we cannot rule out the hypothesis that hypothermia is an effective form of therapy for reducing neurological injuries.

We succeeded in reducing the newborns’ body temperature, at the beginning of and during hypothermia therapy, and successfully performed rewarming. We did not have any potentially serious complication relating to this therapy. The patients had favorable outcomes during the hospitalization period, given the high degree of neurological sequelae in this group of patients.

In most infants with HIE, feeding impairments are present from the neonatal period onwards or started within the first six months.^26^ Most of our patients were discharged with oral feeding, which provided hope, both for us and for the parents, that the neurological outcomes among these patients will be quite satisfactory, with a better quality of life. It is necessary to follow this group of newborns clinically and neurologically, in order to better assess the results from this therapy.

More studies involving hypothermia are needed, with longer exposure, lower temperatures and selective or whole-body hypothermia, and using other related therapies such as erythropoietin, xenon, melatonin, topiramate and stem cell therapy.^27^^,^^28^

## CONCLUSION

We concluded that the use of body hypothermia for neuroprotection among neonates with HIE within daily practice in our hospital did not lead to any problems when performed with a specific protocol and a trained multidisciplinary team. Hypothermia as a form of therapy for asphyxiated newborns was shown to be safe.

## References

[1] Procianoy RS, Silveira RC (2001). Síndrome hopóxico-isquêmica [Hypoxic-ischemic syndrome]. J Pediatr (Rio J).

[2] Kurinczuk JJ, White-Koning M, Badawi N (2010). Epidemiolology of neonatal encephalopathy and hypoxic-ischaemic encephalopathy. Early Hum Dev.

[3] Perlman M, Shah PS (2011). Hypoxic-ischemic encephalopathy: challenges in outcome and prediction. J Pediatr.

[4] Badawi N, Kurinczuk JJ, Keogh JM (1998). Intrapartum risk factors for newborn encephalopathy: the Western Australian case-control study. BMJ.

[5] Gunn AJ (2000). Cerebral hypothermia for prevention of brain injury following perinatal asphyxia. Curr Opin Pediatr.

[6] Wyatt JS, Robertson NJ (2005). Time for a cool head-neuroprotection becomes a reality. Early Hum Dev.

[7] Cowan F, Rutherford M, Groenendaal F (2003). Origin and timing of brain lesions in term infants with neonatal encephalopathy. Lancet.

[8] Gluckman PD, Wyatt JS, Azzopardi D (2005). Selective head cooling with mild systemic hypothermia after neonatal encephalopathy: multicentre randomised trial. Lancet.

[9] Stimbruner G, Mittal RA, Rohlmann F, Muche R, neo.nEURO.network Trial Participants (2010). Systemic hypothermia after neonatal encephalopathy: outcomes of neo.nEURO.network RCT. Pediatrics.

[10] Wyatt JS, Thoresen M (1997). Hypothermia treatment and the newborn. Pediatrics.

[11] Thoresen M, Wyatt J (1997). Keeping a cool head, post-hypoxic hypothermia-an old idea revisited. Acta Paediatr.

[12] Arrich J, Holzer M, Havel C, Müllner M, Herkner H (2012). Hypothermia for neuroprotection in adults after cardiopulmonary resuscitation. Cochrane Database Syst Rev.

[13] Marks K, Shany E, Shelef I, Golan A, Zmora E (2010). Hypothermia: a neuroprotective therapy for neonatal hypoxic ischemic encephalopathy. Isr Med Assoc J.

[14] Shankaran S, Laptook AR, Ehrenkranz RA (2005). Whole-body hypothermia for neonates with hypoxic-ischemic encephalopathy. N Engl J Med.

[15] Tagin MA, Woolcott CG, Vincer MJ, Whyte RK, Stinson DA (2012). Hypothermia for neonatal hypoxic ischemic encephalopathy: an updated systematic review and meta-analysis. Arch Pediatr Adolesc Med.

[16] Jacobs SE, Berg M, Hunt R (2013). Cooling for newborns with hypoxic ischaemic encephalopathy. Cochrane Database Syst Rev.

[17] Eicher DJ, Wagner CL, Katikaneni LP (2005). Moderate hypothermia in neonatal encephalopathy: safety outcomes. Pediatr Neurol.

[18] Cornette L (2012). Therapeutic hypothermia in neonatal asphyxia. Facts Views Vis Obgyn.

[19] Bharadwaj SK, Bhat V (2012). Therapeutic hypothermia using gel packs for term neonates with hypoxic ischaemic encephalopathy in resource-limited settings: a randomized controlled trial. J Trop Pediatr.

[20] Hoque N, Chakkarapani E, Liu X, Thoresen M (2010). A comparison of cooling methods used in therapeutic hypothermia for perinatal asphyxia. Pediatrics.

[21] Lasky RE, Parikh NA, Williams AL, Padhye NS, Shankaran S (2009). Changes in the PQRST intervals and heart rate variability associated with rewarming in two newborns undergoing hypothermia therapy. Neonatology.

[22] Wusthoff CJ, Dlugos DJ, Gutierrez-Colina A (2011). Electrographic seizures during therapeutic hypothermia for neonatal hypoxic-ischemic encephalopathy. J Child Neurol.

[23] Grossmann KR, Tzovla A, Wiberg MK, Hallberg B (2012). Hypothermia-treated infants with hypoxic ischemic encephalopathy (HIE): MRI-findings correlate well with neuromotor-outcome at 12 months. Archives of Disease in Childhood.

[24] Inder TE, Hunt RW, Morley CJ (2004). Randomized trial of systemic hypothermia selectively protects the cortex on MRI in term hypoxic-ischemic encephalopathy. J Pediatr.

[25] Massaro AN, Kadom N, Chang T (2010). Quantitative analysis of magnetic resonance images and neurological outcome in encephalopathic neonates treated with whole-body hypothermia. J Perinatol.

[26] Martinez-Biarge M, Diez-Sebastian J, Wusthoff CJ (2012). Feeding and communication impairments in infants with central grey matter lesions following perinatal hypoxic-ischaemic injury. Eur J Paediatr Neurol.

[27] Johnston MV, Fatemi A, Wilson MA, Northington F (2011). Treatment advances in neonatal neuroprotection and neurointensive care. Lancet Neurol.

[28] Gonzalez FF, Ferriero DM (2009). Neuroprotection in the newborn infant. Clin Perinatol.

